# Family Connects New Orleans: Redefining Fourth Trimester Care

**DOI:** 10.31486/toj.25.0099

**Published:** 2025

**Authors:** Christopher Levi Griffin, Zoë Merritts, Charlene Ho

**Affiliations:** Family Connects New Orleans, Ochsner Clinic Foundation, New Orleans, LA

## THE GAPS IN POSTPARTUM CARE

The early postpartum days can be a time of exciting change, but they do not mark the end of pregnancy-related health risks. In fact, in 2021, 43.3% of pregnancy-related deaths occurred in the 6 weeks following delivery, yet typical practice is still a single 6-week postpartum appointment.^1,2^ The contrast between weekly prenatal monitoring and the sudden silence for up to 6 weeks postpartum is jarring. Why isn’t a similar degree of medical oversight provided for postpartum patients as for antepartum patients, given the increased risk for conditions such as postpartum preeclampsia, postpartum depression, and infection?

Ochsner Health, in partnership with the City of New Orleans, Louisiana, and LCMC Health at Touro Infirmary, has begun to bridge this gap with Family Connects, a universal postpartum home visit program that brings postpartum care in New Orleans closer in line with the most recent guidance from the American College of Obstetricians and Gynecologists (ACOG) for “fourth trimester” care.^2^

## PARADIGM SHIFT FOR THE AMERICAN COLLEGE OF OBSTETRICIANS AND GYNECOLOGISTS

The 2018 “ACOG Committee Opinion No. 736: Optimizing Postpartum Care” marks a major step from historic practice to scientific foundations for the postpartum phase of maternal care.^2^ The opinion addresses the cultural rather than evidential basis for the previous paradigm of postpartum care and recommends a shift to a tailored schedule that begins with contact by a maternal health care provider (eg, physician, advanced practice provider, nurse, midwife, or doula) within 3 weeks of delivery, followed by a comprehensive postpartum visit within 12 weeks.^2^

The ACOG opinion reports that up to 40% of patients nationwide do not attend a formal postpartum visit at all, with even lower percentages among patients of color and populations with limited resources.^2^ While patients delivering at the Ochsner Health flagship birthing center, Ochsner Baptist, are more likely to attend a postpartum visit than the national estimate (82.7% compared to approximately 60%), barriers such as transportation, health literacy, and clinical capacity persist. Racial disparities are also prevalent. For example, among patients delivering at Ochsner Baptist, 87.0% of White patients attended a postpartum visit at any point after delivery, compared to 73.4% of Black/African American patients (data obtained from hospital electronic health records using the Epic SlicerDicer tool [Epic Systems Corporation]). These numbers are noteworthy and reflect the work of a dedicated maternal care system, but room for improvement remains.

The tension between new recommendations and existing barriers to postpartum care raises practical questions. If patients are not attending their 6-week appointment, why would they attend an appointment at 3 weeks? How could already-busy obstetrics clinics accommodate increased appointment demand? What value do these additional points of contact add to the patient experience and the health care system?

The ACOG opinion cites nurse home visits as a viable alternative to clinic visits for addressing these challenges.^2^

## OVERVIEW OF THE FAMILY CONNECTS MODEL

In July 2024, Ochsner Baptist implemented Family Connects,^3^ a postpartum home visit model for patients who deliver at Ochsner Baptist. Compared to means-tested, long-term home visiting programs such as Healthy Start^4^ and other models included in the Maternal, Infant, and Early Childhood Home Visiting Program,^5^ Family Connects stands out as the only *universal* home visiting model, providing services to any family in the catchment area regardless of income or risk levels. This model is reminiscent of large-scale, often government-backed models in countries with a wide range of cultures and income levels in Europe, Africa, and Asia.^6-8^

The Family Connects model was created at the Duke University Center for Child and Family Policy in 2008 and has since expanded to sites across 21 states, ranging from rural community organizations to statewide health departments.^3^ All sites follow the same model of care. Mothers are offered the Family Connects service shortly after giving birth, ideally in person and within the first 24 hours postpartum. An integrated home visit from a registered nurse trained in mother-baby care is scheduled for approximately 3 weeks from the date of delivery. A pre–integrated home visit for targeted needs such as breastfeeding support or blood pressure (BP) checks can also be offered sooner if needed.

During the integrated home visit, the visiting nurse assesses 12 factors across 4 domains (Table 1). The nurse conducts physical assessments of the birth parent and newborn (eg, vital signs, incision inspections, newborn examinations, and growth measurements); provides lactation support; and provides education on topics such as safe sleep, sudden infant death syndrome prevention, household safety, and infant crying patterns. The nurse also administers validated screenings for postpartum depression, substance abuse, and domestic violence. Visits generally conclude with referrals to community organizations or health care providers as indicated. The visits last 1 to 2 hours, allowing for in-depth exploration of concerns not readily addressed during a traditional in-office appointment. Up to 2 follow-up visits may be offered to the family at the discretion of the visiting nurse to conduct additional assessments and/or support connections to community resources.

**Table 1.** Domains Assessed in a Family Connects Integrated Home Visit

Domain	Description
1. Support for health care	Perform physical assessments, ensure appropriate clinical follow-up for mother and baby, coordinate ongoing health care
2. Support for caring for the infant	Advise on childcare plans, encourage bonding, educate about crying patterns
3. Support for a safe home	Support access to resources such as diapers and formula, assess home environment safety
4. Support for parents	Screen for emotional well-being of caregivers, normalize discussion of emotional challenges

## EVIDENCE SUPPORTING UNIVERSAL HOME VISITS

The Family Connects integrated home visits do not replace the need for a comprehensive postpartum appointment with an obstetrician or midwife. Rather, Family Connects visits are intended to fill gaps in the existing postpartum care structures to improve the efficiency of care delivery and to align with the early contact and patient-centered values emphasized in the ACOG guidance. Family Connects integrated home visits can help meet the ACOG recommendation of contact with an obstetric care provider within 3 weeks of delivery without increasing appointment demand at obstetric clinics.

Findings from research on Family Connects support the program's efficacy. The Family Connects model received an evidence-based designation from the Home Visiting Evidence of Effectiveness review process, which evaluates early childhood home visiting models according to criteria set by the US Department of Health & Human Services, with multiple favorable findings in 4 maternal-child health domains (Table 2).^9-13^ Studies of postnatal nurse home visiting programs have demonstrated a reduction in the likelihood of maternal anxiety, a health care cost savings of $3 for every $1 invested in the program, and a reduction in infant emergency medical care utilization of 50% in the first 12 months of life.^12,13^

**Table 2.** Home Visiting Evidence of Effectiveness Domains With Favorable Findings for Family Connects^9-13^

Domain	Outcomes
Child health	• Reduced inpatient overnight hospital stays^11-13^ • Reduced parent-reported child emergency medical care through ages 6 months,^12^ 12 months,^13^ and 24 months^11^
Linkages and referrals	• Increased community connections^12^
Maternal health	• Improvements in postpartum anxiety disorders^12^
Positive parenting practices	• Improvements in positive parenting behaviors^12^

The evidence shows promise for meaningful improvements in maternal and infant health outcomes that Family Connects hopes to replicate and improve upon.

## FAMILY CONNECTS NEW ORLEANS PILOT: LOCAL OUTCOMES

The Family Connects New Orleans sites at Ochsner Baptist and Touro Infirmary provide services at no cost to Orleans Parish residents and are serving as pilot programs for the state of Louisiana with the aim of expanding the model statewide. At Ochsner Baptist, we are studying outcomes for our Family Connects–eligible mothers, including the proportion of patients who receive BP measurements and postpartum depression screenings, as well as the timeliness of those screenings. Early results are promising. Notably, preliminary analysis has shown a reduction in the median time to first BP measurement after discharge from the delivery admission from 28 days to 25 days (Griffin et al, unpublished data, July 2025). While the proportion of patients receiving a postpartum BP measurement did not significantly change based on preliminary data, the reduced time to BP measurement associated with the introduction of Family Connects is encouraging, demonstrating the potential of the program to improve maternal health care utilization. We are also planning a scoping review of all available literature assessing Family Connects programs across the United States with the intention of collating the evidence to better inform decisions about expanding the program at local, state, and national levels.

Staff members at the Tulane University School of Public Health and Tropical Medicine are also conducting an ongoing evaluation of the impacts of Family Connects New Orleans, and their early findings are encouraging. The most recent update revealed that Family Connects participants covered by Medicaid were 12.7% more likely to receive a postpartum visit within 90 days of delivery (*P*<0.01) than nonparticipants, and the observed difference increased to 20% by 270 days (*P*<0.001). The Tulane University evaluation also found reductions in Medicaid spending among Family Connects participants of $202 (*P*=0.79) and $681 (*P*=0.66) for mothers and infants, respectively, at 9 months after delivery (Callison et al, Family Connects New Orleans Evaluation Report, unpublished data, July 2025). While the early results are mixed or have not reached statistical significance, the composite findings suggest improvement that growing sample sizes may help to demonstrate.

## FAMILY CONNECTS AT OCHSNER BAPTIST: LOCAL IMPACTS

July 2025 was the 1-year anniversary of Family Connects at Ochsner Baptist. During the first year of the program, we visited 452 families, conducted 240 follow-up visits, and made more than 200 referrals to community organizations. While the program is still in the implementation phase, Family Connects at Ochsner Baptist is making consistent progress toward receiving full certification from Family Connects International.

Patient-submitted evaluations of the program highlight qualitative domains of added value such as the convenience of receiving care at home and the meaningful connections formed with the visiting nurse. Participants report an exceptionally high satisfaction rate, with 100% of respondents stating they would “probably” or “definitely” recommend Family Connects New Orleans to a friend.

As the program at Ochsner Baptist has grown, we have conducted continual internal assessments and implemented protocols to increase the rate of patients opting in, to decrease the time from discharge to home visit, and to minimize cancellations. These efforts reflect the team's commitment to optimizing performance with the goals of becoming an integral component of postpartum care in New Orleans and serving as a model for statewide expansion.

In their final report submitted in January 2025, the Postpartum Newborn Home Visiting Task Force created by the Louisiana Legislature “strongly recommends comprehensive coverage and reimbursement through Medicaid and private insurance for universal postpartum newborn nurse home visiting services.”^14^ With demonstrated cost savings, improvements in health care engagement, and high patient satisfaction, Family Connects deserves to become part of the standard of postpartum care, improving the fourth trimester experience for every new family in Louisiana.
